# Supplementary figures and images for: Outcomes of patients with active cancers and pre-existing cardiovascular diseases infected with SARS-CoV-2
Source: Cardiooncology. 2023 Oct 6;9:36. doi: 10.1186/s40959-023-00187-w (PMC10557272; doi:10.1186/s40959-023-00187-w)

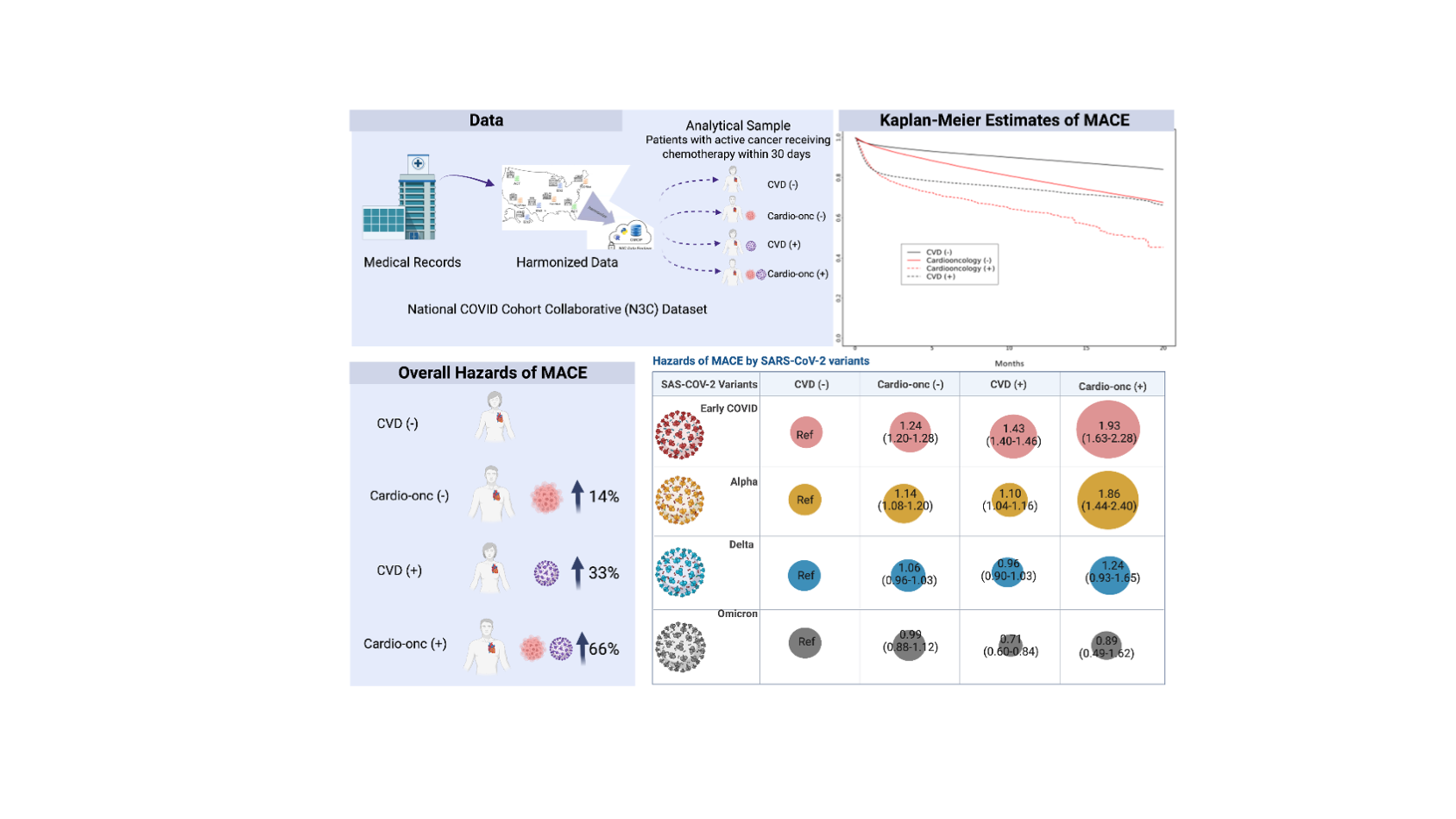

Supplement: Supplementary file 1 — Additional file 1. [file 40959_2023_187_MOESM1_ESM.tiff]
